# Comprehensive Analysis of lncRNA–miRNA– mRNA Network Ascertains Prognostic Factors in Patients with Colon Cancer

**DOI:** 10.1177/1533033819853237

**Published:** 2019-06-03

**Authors:** Zhenzhen Gao, Peng Fu, Zhengyi Yu, Fuxi Zhen, Yanhong Gu

**Affiliations:** 1 Department of Clinical Oncology, The First Affiliated Hospital of Nanjing Medical University, Nanjing, China; 2 Department of Bone Oncology, The Second Affiliated Hospital of Jiaxing University, Jiaxing, China

**Keywords:** colon cancer, microRNAs, long non-coding RNAs, competing endogenous RNA network, overall survival

## Abstract

**Background:**

Non-coding RNAs are competing endogenous RNAs in the occurrence and development of tumorigenesis; numerous microRNAs are aberrantly expressed in colon cancer tissues and play significant roles in oncogenesis development and metastasis. However, large clinical and RNA data are lacking to further confirm the exact role of these RNAs in tumors. This study aimed to ascertain differential RNA expression between colon cancer and normal colon tissues.

**Materials and Methods:**

RNA sequencing and clinical data of patients with colon cancer were procured from The Cancer Genome Atlas database; differentially expressed long non-coding RNA, differentially expressed messenger RNAs, and differentially expressed microRNAs were achieved using the limma package in edgeR to generate competing endogenous RNAs networks. Then, Gene Ontology and Kyoto Encyclopedia of Genes and Genomes enrichment analysis were conducted with ggplot2 package, the Kaplan-Meier survival method was used to predict survival in patients with colon cancer.

**Results:**

In total, 1174 differentially expressed long non-coding RNAs, 2068 differentially expressed messenger RNAs, and 239 differentially expressed microRNAs were generated between 480 colon cancer and 41 normal colon tissue samples. Three competing endogenous RNA networks were established. Gene Ontology analysis indicated that the genes of the up-regulated microRNA network were involved in negative regulation of transcription, DNA-template, and those of down-regulated microRNA network were involved in transforming growth factor β receptor signaling pathways, response to hypoxia, cell migration, while Kyoto Encyclopedia of Genes and Genomes analyses of these networks turned out to be negative. Three long non-coding RNAs (AP004609.1, ARHGEF26-AS1, and LINC00491), 3 microRNAs (miRNA-141, miRNA-216a, and miRNA-193b) and 3 RNAs (ULBP2, PHLPP2, and TPM2) were detected to be associated with prognosis by the Kaplan-Meier survival analysis. Additionally, univariate and multivariate Cox regression analyses showed that the microRNA-216a of the competing endogenous RNA might be an independent prognostic factor in colon cancer.

**Conclusions:**

This study constructed the non-coding RNA-related competing endogenous RNA networks in colon cancer and sheds lights on underlying biomarkers for colon cancer cohorts.

## Introduction

Colorectal cancer has become one of the most common diseases over the past years. Approximately 1.2 million patients worldwide are diagnosed with colorectal cancer every year, while more than 600 000 patients directly or indirectly die from colorectal cancer.^1^ Its morbidity and mortality are very high in China and worldwide. The occurrence of colon cancer (CC) is highly heterogeneous, mostly due to the development of sporadic adenomas.^2-4^ To be precise, about 2.6% to 5.6% of adenomas eventually develop into malignant tumors. The existing treatment methods for colorectal cancer include surgery, radiotherapy, and chemotherapy, and the choice of treatment is related to the stage of the tumor. Postoperative adjuvant radiotherapy and chemotherapy have been the standard treatment for patients with stage III postoperative treatment, and many studies have confirmed that patients with stage II postoperative treatment are in risk of recurrence and metastasis.^5,6^ Approximately half of the patients who require adjuvant therapy eventually die of metastases from colorectal cancer, and metastasis is a major cause of treatment failure.

The competing endogenous RNA (ceRNA) hypothesis came up as a new type of regulatory mechanism between non-coding RNA (ncRNA) and coding messenger RNAs (mRNAs).^7^ Long non-coding RNAs (lncRNAs), over 200 nucleotides long, are reported to participate in transcriptional and post-transcriptional management. MicroRNAs (miRNAs),^8^ with aberrant expression in various tumors, modulate the post-transcriptional RNAs. However, existing studies on the role of miRNAs in CC are controversial, which may be attributed to the small sample size used in these studies. The Cancer Genome Atlas (TCGA) database contains large-sample data, the results of which are true and reliable, and these results are open to all researchers.^9^ This study aimed to generate the differentially expressed miRNAs (DEmiRNAs) using the miRNA data downloaded from the TCGA database. In addition, we determined the relationship between DEmiRNAs and prognosis of patients with CC.

## Materials and Methods

RNA sequencing raw data were downloaded from the TCGA database (https://portal.gdc.cancer.gov/, version 10.1), and 521 individuals with CC were eligible for inclusion. The RNA data of 480 CC and 41 para-carcinoma tissues and clinical data were also acquired from the TCGA database. All data were acquired from the TCGA database. Herein, ethical committee assessment is not required for the study. The clinical information for patients with CC is listed in Table 1.

**Table 1.** The Predictive Values of Clinical Characteristics.

Variables	Patients (N)	Univariate Analysis		Multivariate Analysis	
		HR (95% CI)	*P*	HR (95% CI)	*P*
Age		0.98-1.02	.59	0.91-1.12	.45
<60	56				
≥60	391				
Pathologic stage		1.08-2.14	.02	1.11-2.94	.01
I-II	253				
III-IV	183				
NA	11				
Sex		0.50-1.59	.69	0.58-1.96	.83
Female	212				
Male	235				
BMI		0.89-0.99	.03	0.89-0.99	.04
≤25	77				
>25	153				
NA	218				
Pathology		0.01-5.48	.94	0.01-2.64	.94
Mucinous adenocarcinoma	143				
Papillary adenocarcinoma	2				
NA	1				

Abbreviations: BMI, body mass index; CI, confidence interval; HR, hazards ratio; NA, not available.

### Data Processing and Differential Expression Analysis

The raw RNA sequencing (lncRNA, miRNA, and mRNA) data were corrected, normalized, and its expression calculated. The edgeR package in R (version 3.4.4) statistical software program, on Bioconductor (http://www.bioconductor.org/) was used to identify the differentially expressed mRNAs, lncRNAs and miRNAs between the CC and adjacent-normal colon tissues, and |log2FC| ≥ 2 and *P* < .01 were considered statistically significant. Volcano plots were visualized using the ggplot2 packages in R.

### Construction of the ceRNA Network

The ceRNA network was constructed according to the hypothesis that lncRNAs modulate the activity of mRNAs through being miRNA sponges. On the basis of these theories, the miRcode online tool (http://www.mircode.org) was applied to detect the relation between lncRNAs and miRNAs. At the same time, the MiRDB (http://www.mirdb.org/), picTarBase (https://pictar.mdc.org), and Targetscan (http://www.targetscan.org//) programs were used to predict the target mRNAs of these miRNAs. Finally, lncRNAs, the miRNAs regulated by the lncRNAs, as well as mRNAs, were included to establish the ceRNA network visualized by Cytoscape (version 3.5.2).

### Functional Enrichment Analysis

Gene Ontology (GO) function analyses Kyoto Encyclopedia of Genes and Genomes (KEGG) pathway enrichment analyses were therefore conducted to detect the function of the differentially expressed messenger RNAs (DEmRNAs) in the ceRNA networks with the R clusterProfiler package. Fisher test was used to generate the notable terms, and *P* < .01 was considered to be statistically significant.

### Prediction Model of the CC Prognostic Factors

The Kaplan–Meier (KM) method and log-rank test were applied to detect the relationship among the DEmRNAs, differentially expressed long non-coding RNAs (DElncRNAs) and DEmiRNAs of the ceRNA network and the overall survival (OS) curves of patients with CC were composed meeting the following requirement (*P* < .05). Subsequently, the univariate Cox model and multivariate Cox hazards regression model were imposed to generate the independent prognostic factors among patients with CC.

## Results

### Data Processing and Identification of DEmRNAs, DElncRNAs, and DEmiRNAs

**Figure 1.**
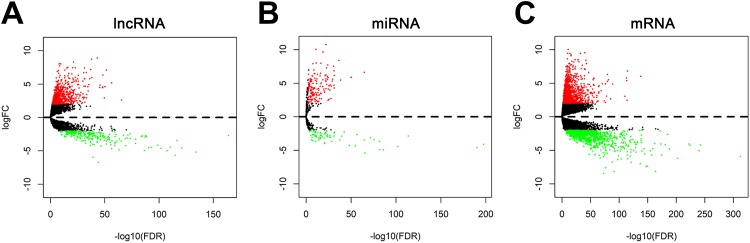
Volcano plots showing the differential expression of RNAs (lncRNAs, miRNAs, and mRNAs) in colon cancer (CC), which were drawn using the R packages ggplot2. X axis reveals log transformed false discovery rate (FDR) values and Y axis implies the mean expression differences of lncRNAs, miRNAs, and mRNAs between 2 groups (|log2FC| ≥ 2 and *P* < .01).

DEmRNAs, DElncRNAs, and DEmiRNAs between CC and adjoining normal colon tissues were determined, with *P* < .01 and |logFC| > 2 as the cut-off criteria. In total, 2067 DEmRNAs containing 1125 upregulated and 942 downregulated, 1174 DElncRNAs containing 933 upregulated, and 241 downregulated, and 239 DEmiRNAs containing 168 upregulated and 71 downregulated were generated between CC and normal mucosa tissues. The volcanic diagrams of DElncRNA, DEmiRNA, and DEmRNA distribution were generated by R. The images are presented in Figure 1.

### Competing Endogenous RNA Network Construction and Function Analysis

**Figure 2.**
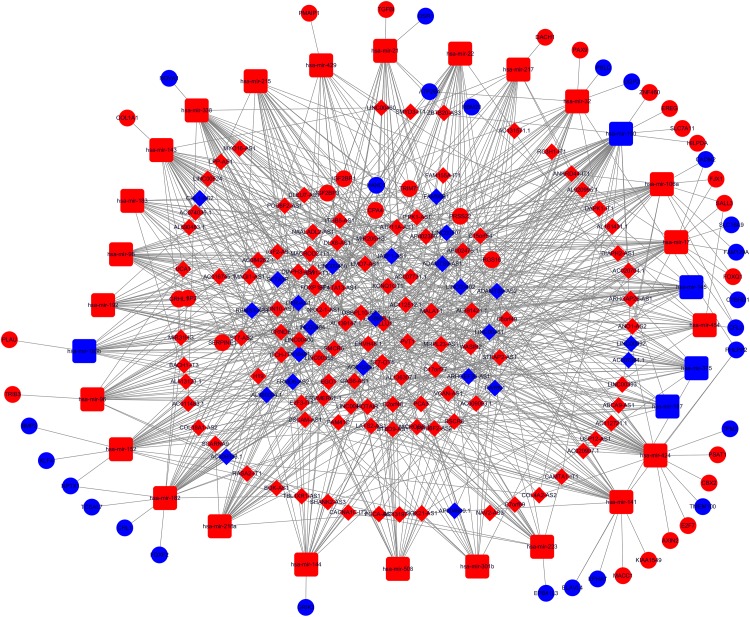
LncRNA-miRNA-mRNA competing endogenous RNA (ceRNA) network of differently expressed RNAs and Gene Ontology (GO) analysis. In the network blue circles = down-regulated mRNAs, red circles = up-regulated mRNAs. Blue quadrates = down-regulated miRNAs, red quadrates = up-regulated miRNAs. Red diamonds = upregulated lncRNAs, and blue diamonds = down-regulated lncRNAs.

**Figure 3.**
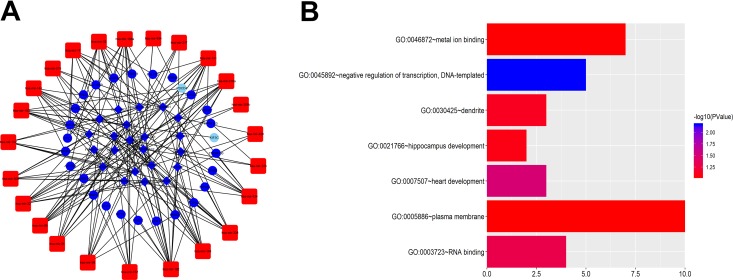
The ceRNA network of up-regulated miRNAs and GO analysis. (A) In the network blue circles = downregulated mRNAs. Red quadrates = up-regulated miRNAs, and blue diamonds = down-regulated lncRNAs. (B) GO results for the target mRNAs of the up-regulated miRNAs ceRNA network. The bar plot indicates the enrichment scores of the significant GO terms (*P* value <.05, gene count ≥3).

**Figure 4.**
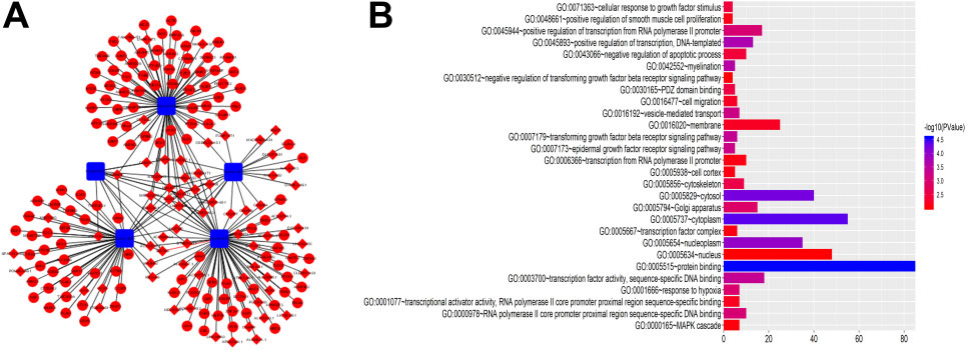
The ceRNA network of down-regulated miRNAs and GO analysis. (A) In the network blue circles = upregulated mRNAs. Red quadrates = down-regulated miRNAs, and blue diamonds = up-regulated lncRNAs. (B) GO results for the target mRNAs of the down-regulated miRNAs ceRNA network. The bar plot indicates the enrichment scores of the significant GO terms (*P* value <.01, gene count ≥3).

**Figure 5.**
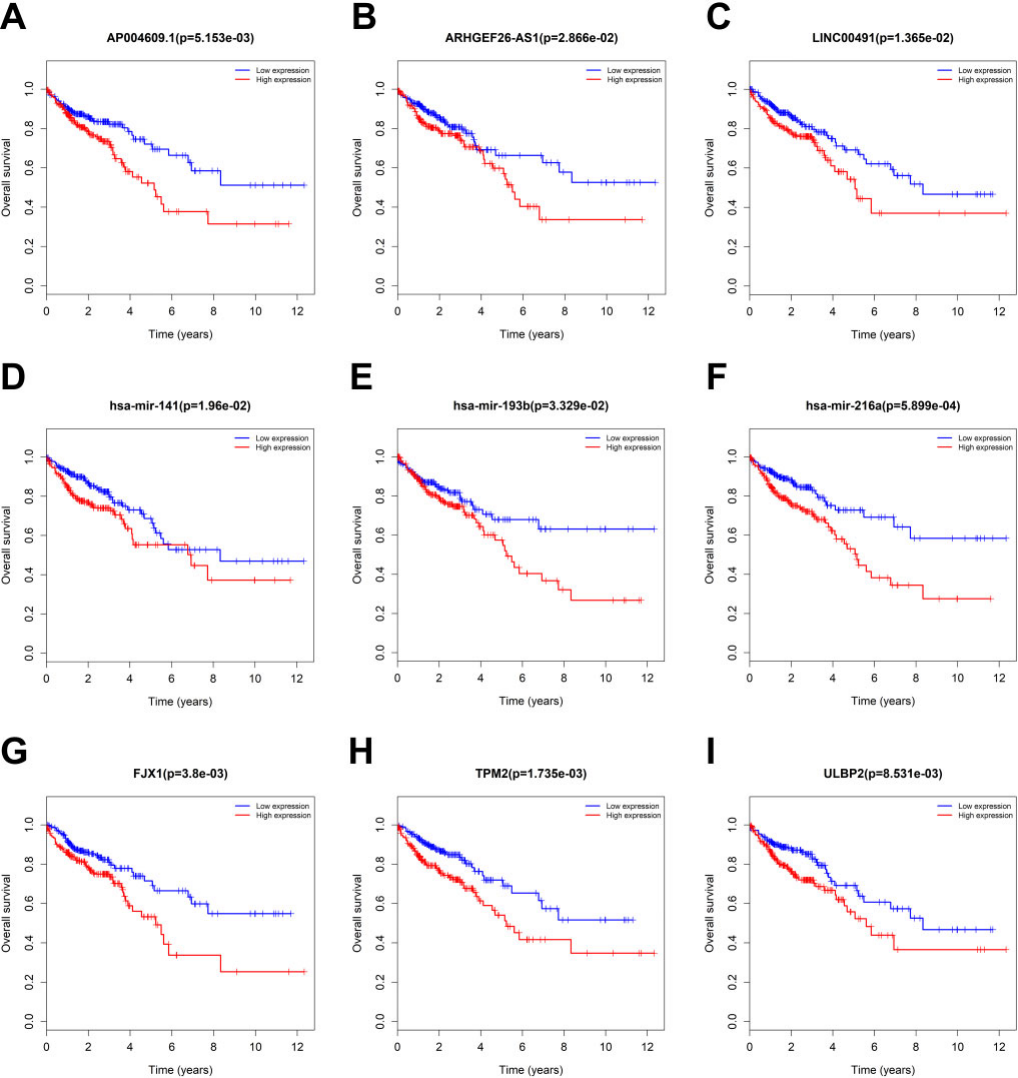
Kaplan–Meier survival curves for (A-C) DElncRNAs, (D-F) DEmiRNAs, and (G-I) DEmRNAs associated with overall survival (OS) of the patients with CC.

The DElncRNAs interacting with DEmiRNA, DEmRNA targeted by DEmiRNA screened using the online database, and DEmiRNA were used to construct the ceRNA network. The relationships between the 1174 DElncRNAs and the 239 DEmiRNAs were firstly assessed, and the functional analysis of these mRNAs was shown in Figure S1, these genes mainly participate in neuroactive ligand-receptor interaction, bile secretion, fat digestion and absorption, as listed in Table S3. The miRcode tool was used to detect the potential relationship, and 29 CC-specific DEmiRNAs that constructively target 131 CC-specific DElncRNAs were then generated. The MiRDB, miRTarBase and Targetscan projects were then used to determine the relationship between the 239 DEmiRNAs and the 2067 DEmRNAs, and predict the mRNA targets of miRNAs. The ceRNA network comprising results of 131 DElncRNAs, 22 CC-specific miRNAs and 54 CC-specific mRNAs was established (Figure 2). On the basis of up-regulated miRNAs are accompanied by down-regulated lncRNAs and mRNAs,^10^ the other 2 ceRNA networks containing upregulated 24 miRNAs, 22 downregulated lncRNAs and 6 mRNAs(Figure 3A) and 5 downregulated miRNAs, 68 lncRNAs, 6 mRNAs were also established and visualized using Cytoscape 3.5.2 (Figure 4A).

Additionally, we performed GO analysis and KEGG pathway enrichment analysis on the target mRNAs of miRNA in the ceRNA networks and selected meaningful GO entries but none meaningful pathway terms. The results of GO analysis are shown in Figure 2B, Figure 3B and Figure 4B. The KEGG pathway analysis revealed that mRNAs of the 3 ceRNA networks were not significantly enriched. To further enhance the bioinformatics analysis reliability, the overlapping genes were analyzed by the Discovery bioinformatics tool (https://david.ncifcrf.gov/). Discovery bioinformatics tool is a web-based online bioinformatics resource that aims to provide a comprehensive set of functional annotation tools for the researchers to understand the biological mechanisms associated with large lists of genes/proteins. Gene Ontology analyses were then performed for the target genes. The *P* value <.01 and gene count ≥3 were set as the cut-off criteria. The genes of the up-regulated miRNA network participated in negative regulation of transcription, DNA-template, and those of down-regulated ones were involved in transforming growth factor β receptor signaling pathways, response to hypoxia, cell migration and positive regulation of transcription from RNA polymerase II promoter, while KEGG pathway enrichment analyses of these networks turned out to be negative. Herein, these GO terms are associated with CC pathogenesis and prognosis.

### Correlations Between CC-Specific Differentially Expressed RNAs and OS

The ceRNA networks are receiving increasing attention, and the function of the differentially expressed RNAs (DERNAs) in the network is detected by the GO analysis; thus, it is suggested that these DERNAs play an important role in the invasion and metastasis of CC. On the basis of these observations, we analyzed the connections between DERNAs and OS of CC by KM analysis. In total, 3 DElncRNAs (ARHGEF26-AS1, AP004609.1, and LINC00491; Figure 5A-C), 3 DEmiRNAs (hsa-miR-141, hsa-miR-216a, and hsa-miR-193b; Figure 5D-F), and 3 DEmRNA (TPM2, FJX1, and ULBP2; Figure 5G-I) were found to be related to OS (*P* < .05).

To further determine the relationship between these DERNAs and OS, all the above DERNAs were then fitted into the multivariate Cox regression model, which indicated that only microRNA-216a had a significant prognostic value in CC (Tables 2 [Table table3-1533033819853237]-4), and then a prognostic model containing miR-216a was established. A risk score analysis of the RNA was performed for each patient, and based on the risk scores, the patients were assigned into the “low risk” and “high risk” groups. It turns out that the OS of the high-risk group is worse than that of the low-risk group (Figure S2a). The 5-year survival correlation of the miR216a signature was analyzed using a receiver operating characteristic curve (ROC) curve (Figure S2b); at the same time, the area under the curve of the miRNA-216a signature was 0.756. Taken together, the findings of this study indicate that miRNA-216a may be an independent prognostic marker in CC.

**Table 2.** Multivariate Analyses of DElncRNAs and DEmiRNAs.

	coef	exp(coef)	se(coef)	*z*	*P*
LINC02474	0.1075	1.1135	0.0382	2.81	.0049
AC020891.2	0.2575	1.2936	0.0854	3.02	.0026
AC010789.1	0.0925	1.0969	0.0569	1.63	.1038
AC093895.1	0.1207	1.1283	0.0562	2.15	.0318
AC105118.1	0.2525	1.2873	0.1066	2.37	.0179
LINC00626	0.2393	1.2704	0.0901	2.66	.0079
AC109439.2	−0.1782	0.8368	0.1082	−1.65	.0997
AC002076.1	0.2817	1.3254	0.108	2.61	.0091
AC112178.1	0.4669	1.595	0.1653	2.82	.0047
hsa-mir-32	0.3235	1.382	0.1643	1.97	.0489
hsa-mir-3917	−0.2218	0.8011	0.106	−2.09	.0363
hsa-mir-144	−0.1652	0.8477	0.0887	−1.86	.0624
hsa-mir-495	0.2974	1.3464	0.1408	2.11	.0346
hsa-mir-3677	−0.2556	0.7744	0.1077	−2.37	.0176
hsa-mir-216a	0.2835	1.3278	0.0944	3	.0027
hsa-mir-548f-1	0.1813	1.1987	0.0719	2.52	.0117
hsa-mir-3189	−0.2348	0.7907	0.1391	−1.69	.0915
hsa-mir-6854	−0.3689	0.6915	0.1203	−3.07	.0022
hsa-mir-552	−0.0742	0.9285	0.0489	−1.52	.1291

**Table 3. table3-1533033819853237:** Univariate Analyses of DElncRNAs.

Gene	HR	*z*	*P* Value
AC112178.1	1.599896	5.215038	1.84E-07
AC093895.1	1.255078	4.103347	4.07E-05
LINC02474	1.139723	3.862502	.000112
AC105118.1	1.459718	3.773514	.000161
AC002076.1	1.465898	3.71396	.000204
AC020891.2	1.327467	3.627038	.000287
LINC02257	1.237372	3.380771	.000723
AC010789.1	1.195537	3.252325	.001145
TMEM132D-AS1	1.121818	3.069367	.002145
AL592043.1	1.226904	2.919669	.003504
LINC00626	1.270428	2.918753	.003514
AC084816.1	1.391183	2.905482	.003667
AC109439.2	1.195422	2.827137	.004697
AC128689.1	1.199056	2.805635	.005022
AC079612.1	0.733282	−2.80065	.0051
HOTAIR	1.117985	2.798841	.005129
AC073283.1	1.208907	2.790909	.005256
AC016027.1	0.69313	−2.74065	.006132
LINC01354	1.237841	2.729518	.006343
AC012531.1	1.163452	2.72888	.006355
LINC01419	1.196575	2.728512	.006362
LINC01980	1.145263	2.717505	.006578
AC003101.2	1.282833	2.647039	.00812
AC083967.1	1.207538	2.63756	.00835
AL590483.1	0.829325	−2.63174	.008495
AC067930.5	1.196168	2.618678	.008827
AC024581.1	1.304851	2.604255	.009207
RBAKDN	1.222634	2.601895	.009271
AC011840.1	1.180715	2.585287	.00973
TSPEAR-AS2	1.148914	2.580129	.009876

Abbreviation: HR, hazards ratio.

**Table 4.** Univariate Analyses of DEmiRNAs.

Gene	HR	*z*	*P* Value
hsa-mir-3677	0.73348	−3.315	.000916
hsa-mir-3189	0.672188	−3.09401	.001975
hsa-mir-216a	1.322105	3.091749	.00199
hsa-mir-6854	0.719463	−2.9269	.003424
hsa-mir-4999	1.375342	2.91512	.003556
hsa-mir-187	1.200106	2.865329	.004166
hsa-mir-149	1.271881	2.66238	.007759
hsa-mir-126	1.471585	2.540097	.011082
hsa-mir-3917	0.785196	−2.50402	.012279
hsa-mir-144	0.809247	−2.43036	.015084
hsa-mir-495	1.358119	2.21251	.026931
hsa-mir-32	1.37298	2.178858	.029342
hsa-mir-376a-1	1.251262	2.136697	.032623
hsa-mir-328	1.327899	2.134753	.032781
hsa-mir-337	1.324207	2.074102	.03807
hsa-mir-7641-1	0.80183	−2.06894	.038551
hsa-mir-136	1.237593	2.059875	.03941
hsa-mir-486-2	0.833528	−2.04339	.041013
hsa-mir-217	1.17929	2.022622	.043112
hsa-mir-548f-1	1.146861	2.001833	.045303
hsa-mir-552	0.906734	−1.98734	.046885
hsa-mir-26b	1.38726	1.985815	.047054

Abbreviation: HR, hazards ratio.

## Discussion

Colon cancer is one of the common malignant tumors of the digestive tract, and its high prevalence rate has been a major problem in developed countries in Europe and the United States.^11,12^ Increasing studies confirmed that ncRNAs have a definite regulatory role in the occurrence of CC5. These ncRNAs are tightly involved in the occurrence, metastasis of tumors, and drug resistance.^13^ Moreover, many studies have demonstrated that miRNAs play an important role in post-transcriptional modification of some mRNAs, which then regulate the function of key molecules, thereby affecting cell behaviors.^14,15^ Along with the development of science and technology, tens of thousands of genes have been reported to associate with the invasion and metastasis of tumors. However, these genes cannot be the key target molecules for tumor treatment, because they do not exist independently but are present in a common environment where there must be interactions between them.^16-19^ This study aimed to discover the RNAs participating in the occurrence and development of CC, further affecting the prognosis of patients with CC by analyzing the ceRNA networks, and then further evaluate the prognosis of RNAs by a Cox regression analysis in order to determine exactly the molecules that would act as a guide for clinical treatment.^20,21^

In the present study, we analyzed high-throughput data, and identified that 2 upregulated miRNAs (miR-141, miR-216a, and miR-193b), 3 lncRNAs (ARHGEF26-AS1, AP004609.1, and LINC00491), and 3 mRNAs (TPM2, FJX1, and ULBP2) were associated with the clinical outcome of patients with CC. Among these prognostic genes, ULBP2 is a cell-surface glycoprotein located on human chromosome 6.^22^ The structure of this molecule is different from class major histocompatibility complex (MHC)class I molecules, which does not contain domain α3, but only α1,2, and is combined with the cell membrane by glycophosphatidylinositol (GPI). Moreover, it can also serve as a ligand of NKG2D, which can activate the human immune system to kill tumors and avoid damaging the normal tissues of the body.^23^ PHLPP2, a kind of phosphatases rich in leucines, is a member of protein phosphatase PHLPP and an important regulator of protein kinase B (AKT), which can dephosphorylate AKT and thus inhibit tumor development. Additionally,^24^ the heterozygous loss of PHLPP2 has been reported to be associated with reversing the development of breast cancer, liver cancer, and ovarian cancer.^25,26^ TPM2, one of the tropomyosin proteins, participates in the regulation of muscle contraction by forming complexes with actin and troponin, and is involved in cellular biological activities (cell invasion, etc).^27^ In addition, more than 40 subtypes of this type of tropomyosin are derived from the 4 categories (TPM1-4). Tm1 subtype is one of the 2 mutants of TPM2 gene, and studies have confirmed that Tm1 is low expressed in prostate cancer,^28^ yet the exact molecular function of the genes remains unknown. Existing studies have confirmed that the occurrence, development, and metastasis of CC are closely related to the PI3K/AKT and Wnt signaling pathways, among others. DERNA GO analysis in the ceRNA networks of this study indicated that these DEmRNAs are mainly involved in transcription factor regulation, angiogenesis, and epithelial mesenchymal transformation, and these biological activities are closely related to the invasion and metastasis of CC, which provides a new perspective for clinical treatment of CC.

LncRNA-miRNA-mRNA ceRNA networks have been reported in various tumors^29^, such as renal clear cell carcinoma,^30^ prostate cancer,^31^ endometrial cancer^32^ and so on. In some of these studies, the RNA-sequence data were obtained from *in vitro* models. Other studies constructed ceRNA network using TCGA database,^30^ then confirm key markers through KEGG pathway enrichment and GO analysis, while no further survival analysis was performed in most of the researches.^31^ We not only established the ceRNA networks, but found prognostic factors based on it, which were further verified by univariate and multivariate regression models.

A multivariate Cox regression model confirmed miR-216a to be the independent prognostic factor among all differential RNAs, and risk values were established through multivariate analysis to divide clinical patients into high-risk groups and low-risk groups. A further ROC curve analysis also suggested that mir-216a was of great significance for the prognosis of CC.

In conclusion, we constructed 3 ceRNA networks and identified various RNAs as potential prognostic predictors for patients with CC. Nevertheless, further *in vitro* and *in vivo* experiments are necessary to validate our findings, and further investigation into the functions is also required to explore the molecular mechanism of markers in CC progression.

## Supplemental Material

Supplemental Material, TableS1 - Comprehensive Analysis of lncRNA–miRNA– mRNA Network Ascertains Prognostic Factors in Patients with Colon Cancer Click here for additional data file. Supplemental Material, TableS1 for Comprehensive Analysis of lncRNA–miRNA– mRNA Network Ascertains Prognostic Factors in Patients with Colon Cancer by Zhenzhen Gao, Peng Fu, Zhengyi Yu, Fuxi Zhen and Yanhong Gu in Technology in Cancer Research & Treatment

## Abbreviations

CC: colon cancer
ceRNA: competing endogenous RNA
DElncRNA: differentially expressed long non-coding RNA
DEmRNA: differentially expressed messenger RNA
DEmiRNA: differentially expressed microRNA
GO: Gene Ontology
KEGG: Kyoto Encyclopedia of Genes and Genomes
lncRNA: long non-coding RNA
ncRNA: non-coding RNA
mRNA: messenger RNA
miRNA: microRNA
OS: overall survival
TCGA: The Cancer Genome Atlas.

## References

[1] Siegel RL Miller KD Jemal A Cancer statistics, 2018. CA Cancer J Clin. 2018;68(1):7–30. 29313949 10.3322/caac.21442

[2] Hu HF Xu WW Wang Y Comparative proteomics analysis identifies cdc42-cdc42bpa signaling as prognostic biomarker and therapeutic target for colon cancer invasion. J Proteome Res. 2018;17(1):265–275. 29072916 10.1021/acs.jproteome.7b00550

[3] Fang M Li Y Huang K IL33 Promotes colon cancer cell stemness via JNK activation and macrophage recruitment. Cancer Res. 2017;77(10):2735–2745. 28249897 10.1158/0008-5472.CAN-16-1602 PMC5760170

[4] Babaei M Balavarca Y Jansen L Administration of adjuvant chemotherapy for stage ii-iii colon cancer patients: an European population-based study. Int J Cancer. 2018;142(7):1480–1489. 29159866 10.1002/ijc.31168 PMC6002808

[5] Al-Haidari AA Syk I Thorlacius H MiR-155-5p positively regulates CCL17-induced colon cancer cell migration by targeting RhoA. Oncotarget. 2017;8(9):14887–14896. 28146427 10.18632/oncotarget.14841 PMC5362452

[6] Li XX Zhang HS Xu YM Knockdown of IRE1alpha inhibits colonic tumorigenesis through decreasing beta-catenin and IRE1alpha targeting suppresses colon cancer cells. Oncogene. 2017;36(48):6738–6746. 28825721 10.1038/onc.2017.284

[7] Yang R Xing L Wang M Chi H Zhang L Chen J Comprehensive analysis of differentially expressed profiles of lncRNAs/mRNAs and miRNAs with associated ceRNA networks in triple-negative breast cancer. Cell Physiol Biochemi. 2018;50(2):473–488. 10.1159/000494162 30308479

[8] Zhang Z Qian W Wang S Analysis of lncRNA-associated ceRNA network reveals potential lncRNA biomarkers in human colon adenocarcinoma. Cell Physiol Biochem. 2018;49(5):1778–1791. 30231249 10.1159/000493623

[9] Song YX Sun JX Zhao JH Non-coding RNAs participate in the regulatory network of CLDN4 via ceRNA mediated miRNA evasion. Nat Commun. 2017;8(1):289. 28819095 10.1038/s41467-017-00304-1 PMC5561086

[10] Cesana M Cacchiarelli D Legnini I A long noncoding RNA controls muscle differentiation by functioning as a competing endogenous RNA. Cell. 2011;147(2):358–369. 22000014 10.1016/j.cell.2011.09.028 PMC3234495

[11] Ma ZG Ma R Xiao XL Azo polymeric micelles designed for colon-targeted dimethyl fumarate delivery for colon cancer therapy. Acta Biomater. 2016;44:323–331. 27544813 10.1016/j.actbio.2016.08.021

[12] Chang YC Su CY Chen MH Chen WS Chen CL Hsiao M Secretory RAB GTPase 3C modulates IL6-STAT3 pathway to promote colon cancer metastasis and is associated with poor prognosis. Mol Cancer. 2017;16(1):135. 28784136 10.1186/s12943-017-0687-7 PMC5547507

[13] Fu XM Guo W Li N The expression and function of long noncoding RNA lncRNA-ATB in papillary thyroid cancer. Eur Rev Med Pharmacol Sci. 2017;21(14):3239–3246. 28770959

[14] Rak B Marczewska JM Wlodarski P The role of microRNAs in endometrial cancer and influence on future therapy: focusing on miRNA-21. Eur J Gynaecol Oncol. 2016;37(5):599–603. 29786994

[15] Wei YT Guo DW Hou XZ Jiang DQ Mirna-223 suppresses FOXO1 and functions as a potential tumor marker in breast cancer. Cell Mol Biol (Noisy-le-grand). 2017;63(5):113–118. 28719355 10.14715/cmb/2017.63.5.21

[16] Misawa A Takayama K Urano T Inoue S Androgen-induced long noncoding RNA (lncRNA) SOCS2-AS1 promotes cell growth and inhibits apoptosis in prostate cancer cells. J Biol Chem. 2016;291(34):17861–17880. 27342777 10.1074/jbc.M116.718536 PMC5016176

[17] Jing H Qu X Liu L Xia H A novel long noncoding RNA (lncRNA), LL22NC03-N64E9.1, promotes the proliferation of lung cancer cells and is a potential prognostic molecular biomarker for lung cancer. Med Sci Monit. 2018;24:4317–4323. 29935018 10.12659/MSM.908359 PMC6047588

[18] Karreth FA Pandolfi PP ceRNA cross-talk in cancer: when ce-bling rivalries go awry. Cancer Discov. 2013;3(10):1113–1121. 24072616 10.1158/2159-8290.CD-13-0202 PMC3801300

[19] Fan CN Ma L Liu N Systematic analysis of lncRNA-miRNA-mRNA competing endogenous RNA network identifies four-lncRNA signature as a prognostic biomarker for breast cancer. J Transl Med. 2018;16(1):264. 30261893 10.1186/s12967-018-1640-2 PMC6161429

[20] Wang J Wang Y Zhou R Host long noncoding RNA lncRNA-PAAN regulates the replication of influenza A virus. Viruses. 2018;10(6):E330. 29914164 10.3390/v10060330 PMC6024364

[21] Yuan W Guo YQ Li XY MicroRNA-126 inhibits colon cancer cell proliferation and invasion by targeting the chemokine (C-X-C motif) receptor 4 and Ras homolog gene family, member A, signaling pathway. Oncotarget. 2016;7(37):60230–60244. 27517626 10.18632/oncotarget.11176 PMC5312381

[22] Leung WH Vong QP Lin W PRL-3 mediates the protein maturation of ULBP2 by regulating the tyrosine phosphorylation of HSP60. J Immunol. 2015;194(6):2930–2941. 25687758 10.4049/jimmunol.1400817 PMC4355089

[23] Champsaur M Lanier LL Effect of NKG2D ligand expression on host immune responses. Immunol Rev. 2010;235(1):267–285. 20536569 10.1111/j.0105-2896.2010.00893.x PMC2885032

[24] Peng M Wang J Zhang D PHLPP2 stabilization by p27 mediates its inhibition of bladder cancer invasion by promoting autophagic degradation of MMP2 protein. Oncogene 2018 doi:10.1038/s41388-018-0374-1 10.1038/s41388-018-0374-1 PMC6202328 29930380

[25] Mathur A Rizvi F Kakkar P PHLPP2 down regulation influences nuclear Nrf2 stability via Akt-1/Gsk3beta/Fyn kinase axis in acetaminophen induced oxidative renal toxicity: protection accorded by morin. Food Chem Toxicol. 2016;89:19–31. 26767949 10.1016/j.fct.2016.01.001

[26] Liao Y Deng Y Liu J MiR-760 overexpression promotes proliferation in ovarian cancer by downregulation of PHLPP2 expression. Gynecol Oncol. 2016;143(3):655–663. 27726922 10.1016/j.ygyno.2016.09.010

[27] Hsu WL Ma YL Liu YC Lee EHY Smad4 SUMOylation is essential for memory formation through upregulation of the skeletal myopathy gene TPM2. BMC Biol. 2017;15(1):112. 29183317 10.1186/s12915-017-0452-9 PMC5706330

[28] Hsu WL Ma YL Liu YC Lee EHY Hypoxia-induced TPM2 methylation is associated with chemoresistance and poor prognosis in breast cancer. Cell Physiol Biochem. 2018, 45(2):692–705. 29414807 10.1159/000487162

[29] Li DY Chen WJ Luo L Prospective lncRNA-miRNA-mRNA regulatory network of long non-coding RNA LINC00968 in non-small cell lung cancer A549 cells: a miRNA microarray and bioinformatics investigation. Int J Mol Med. 2017;40(6):1895–1906. 29039552 10.3892/ijmm.2017.3187

[30] Zhu H Lu J Zhao H Functional long noncoding RNAs (lncRNAs) in clear cell kidney carcinoma revealed by reconstruction and comprehensive analysis of the lncRNA-miRNA-mRNA regulatory network. Med Sci Monit. 2018;24:8250–8263. 30444862 10.12659/MSM.910773 PMC6251074

[31] He JH Han ZP Zou MX Analyzing the LncRNA, miRNA, and mRNA regulatory network in prostate cancer with bioinformatics software. J Comput Biol. 2018;25(2):146–157. 28836827 10.1089/cmb.2016.0093

[32] Liu C Zhang YH Deng Q Cancer-related triplets of mRNA-lncRNA-miRNA revealed by integrative network in uterine corpus endometrial carcinoma. Biomed Res Int. 2017;2017:3859582. 28280730 10.1155/2017/3859582 PMC5320387

