# Supplementary material for: Comprehensive Analysis of lncRNA–miRNA– mRNA Network Ascertains Prognostic Factors in Patients with Colon Cancer
Source: Technol Cancer Res Treat. 2019 Jun 3;18:1533033819853237. doi: 10.1177/1533033819853237 (PMC6552362; doi:10.1177/1533033819853237)
Supplement: Supplemental Material, TableS1 - Comprehensive Analysis of lncRNA–miRNA– mRNA Network Ascertains Prognostic Factors in Patients with Colon Cancer [file TableS1.pdf]

GO results for differentially expressed genes.

Term	Count	PValue	Genes
GO:0005576~extracellular region	347	1.42E-42	EDN3, MASP1, S100A7, EDN2, NOTUM, LUZP2, DNASE1L2, DNASE1L3, SHH, OGN, DSPP, APOB, AGTR2, SERPINE1, COL12A1, IFNK, GUCA2A, MUC15, CFD, BRINP3, COL10A1, WFDC10A, MATN3, WFDC10B, TMEFF2, STC2, NODAL, COLEC10, F7, DHRS7C, CLEC4M, PRELP, WFDC8, VEGFD, CD163L1, PPBP, F5, TMPRSS11D, TMPRSS11E, F2, IL36B, PLA2G2C, TMPRSS11A, ADAMTS2, PLA2G2F, IBSP, ODAM, ENPP6, IL36G, ACP7, GAST, ESM1, FAM19A4, ITGBL1, AHSG, IL17C, METTL24, IL17A, IL23A, INS, IL17F, SPP1, MLN, BMP3, NTF4, IGFL4, HBA2, MUCL1, LAMA1, CEL, NPY, PENK, SFRP1, NPW, PSORS1C2, SFRP4, TREML4, IGFL2, PAPPA2, IGFL3, MUC5AC, PYY, BMP7, PLAU, BMP5, NXPE1, TLL1, FGF8, MTRNR2L1, ASGR1, KISS1, CGREF1, MMP20, TNFRSF11B, SERPINA7, ERFE, CREG2, SERPINA4, SEMA3E, GPX3, COL11A1, FGF3, GHR, SPACA3, ELANE, SAA4, GIF, PSG1, IL6R, MMP13, FLNA, MMP11, SLIT3, INHBB, MMP10, INHBA, PSG9, CHRDL1, BGN, PSG7, EREG, PSG6, PSG5, OTOG, SERPINB2, SCGB3A2,

ADAM12, PLA2G3, PRSS48, PLA2G5, CXCL1, CER1, TG, CSF2, C7, CXCL5, CXCL3, CXCL2, JCHAIN, CXCL8, NMB, CXCL6, CCL28, IGSF10, CCL23, COL7A1, COL6A6, CCL21, COL6A5, THBS2, HGFAC, NMU, WNT8B, LCN15, HAPLN1, LCN12, CCL19, IGF2, LCN10, DKK4, ORM1, CCL13, WNT7B, CCL14, COL19A1, DKK1, CLEC3B, MAPK15, LRP8, CMA1, SERPIND1, PRSS22, WNT7A, ORM2, ENHO, FGF19, FDCSP, CGB5, CNDP1, WNT3A, OBP2A, F13A1, MMP8, OBP2B, MMP7, CGB7, MMP3, CXCL11, MMRN1, CXCL12, GDNF, MMP1, PSG11, IL11, APOA4, AADACL2, WNT2, AZGP1, APOA2, APOA1, WNT3, PGLYRP4, PGLYRP3, TGFBI, EPPIN, LGI4, REG3G, LGI1, IL1A, SEMG1, BPIFA1, SCUBE2, SEMG2, FGF23, FGF21, FGF20, TCN1, PCOLCE2, UCN2, CST5, ANOS1, MFAP2, PAEP, COL1A1, MFAP4, MFAP5, FGFR2, BPIFB1, PPY, HIST1H4L, CLU, GREM2, TIMP1, ANGPTL7, SAA2-SAA4, CLPSL2, FOLR2, FGA, HIST1H4A, BCHE, FGB, HIST1H4B, APELA, CLPSL1, APOC3, GALP, SFTA3, HIST1H4E, SFTA2, SFTPC, HIST1H4F, HIST1H4C, HIST1H4D, DEFB1, ANGPTL5, SFTPB,

GO:0005615~extracellular space	301	9.97E-40	PRB2, HIST1H3J, DAND5, PRB1, PRB4, DMP1, EPHX3, SELENOP, NTN1, MUC6, HIST2H3D, LCN2, LCN1, CXCL17, OMD, S100B, LCN6, HIST1H3A, HIST1H3B, HIST1H3C, WIF1, HIST1H3D, HIST1H3F, SST, HABP2, HIST1H3I, PNLIP, INSL5, NXPH4, SLURP1, CORIN, REG4, CRP, RSPO4, WISP3, SPINT4, RSPO2, CNTNAP3, KLK10, NRG2, APLN, ITIH6, NXPH1, LAIR2, INHA, GRP, OSM, AMH, SIGLEC6, CHST9, CA6, CEMIP, CNTN4, WNT11, TREM1, CTSG, VIP, HCRT, TSPEAR, PRSS1, ISM2, C1QTNF9, C1QTNF7, CALCA, CALCB, C1QTNF8, COL9A3, NPTX2, COMP, PRSS2, REN, SCG3, ACAN, HBB, KLK6, KLK7, VSTM2A, PLAC1, DHRS11, PLAC9, A2ML1, COL4A6, GCG, SCGN, PNOC, C5ORF38, DSC3, IGFBP1, GDF15, ADAMDEC1, LIPF EDN3, CTHRC1, LYPD3, GLDN, MASP1, PGC, EDN2, LYPD8, CRHBP, SELENBP1, SHH, OGN, ACTG2, APOB, IFNE, FAP, PPP1R1A, HIST1H2BI, HIST1H2BJ, SERPINE1, CCBE1, COL12A1, IFNK, CFD, VNN3, INA, CELA3B, CLCA1, STC2, ADAMTS20, NODAL, PKHD1L1, F7, PRELP, SCGB2A1, VEGFD, CD36, PPBP, F5, TACSTD2, F2, IL36B, STC1, IBSP, ODAM, IL36G, SERPINA10,

GAST, AHSG, IL17C, IL17A, INS, IL17F, OLFM3, SPP1, BMP3, IGFL4, IL1RN, CHI3L1, KRT31, HILPDA, KRT34, KRT35, SFRP5, LAMA1, CEL, LGALS7B, NPY, SFRP1, SRPX2, SFRP4, FJX1, IGFL1, MUC5AC, PYY, BMP7, PLAU, BMP5, CSTL1, FGF8, PRH2, SPINK2, ANPEP, KISS1, SERPINA9, MMP20, TNFRSF11B, SERPINA7, SAA2, SMPDL3A, SCRG1, ERFE, SERPINA4, SEMA3E, GPX3, PRSS33, DPEP1, VWA2, GHR, SPACA3, EGFL6, ELANE, CPXM2, GIF, SAA4, IL6R, MMP13, SLIT3, INHBB, MMP10, EREG, SERPINB5, SERPINB7, OTOG, LYZL4, SERPINB2, SERPINB4, SERPINB3, EPYC, PLA2G3, CXCL1, CER1, TG, CSF2, CXCL5, CXCL3, CXCL2, JCHAIN, CXCL8, PSAPL1, CXCL6, CCL28, CCL26, CPN1, PCSK2, PCSK1, CCL23, COL7A1, CCL21, VWC2, PCSK9, SERPINB13, PCSK5, HGFAC, WNT8B, CES3, CES2, HIST1H2BE, HIST1H2BF, NLGN1, CCL19, IGF2, AFP, ORM1, CCL13, WNT7B, CCL14, DKK1, CLEC3B, EPGN, SERPIND1, WNT7A, ORM2, CMTM5, WNT3A, MMP8, MMP7, DLK1, MMP3, CXCL11, CXCL12, IL11, WNT2, APOA4, AZGP1, REG3A, APOA1, WNT3, SPX, TGFBI, TDGF1,

			MS4A1, EPPIN, LGI4, CPA1, LGI1, KRT83, IL1A, SEMG1, BPIFA1, SPARCL1, SCUBE1, SEMG2, CST2, FGF23, CST1, FGF21, TCN1, UCN2, CBLN2, CST6, CST4, CST5, CARTPT, ANOS1, RELN, COL1A1, XDH, ACTBL2, BPIFB1, PPY, BPIFB2, MSMB, CLU, DSCAML1, CCL8, GREM2, ABI3BP, TIMP1, FGA, FGB, APELA, MSLN, BTN1A1, APOC3, TFF2, SFTPC, ANGPTL1, ANGPTL3, DEFB1, SFTPB, DAND5, CPA4, LPO, UCN, SELENOP, C2ORF40, LCN2, LCN1, CXCL17, OMD, S100B, SPTBN2, SST, HABP2, INSL4, SLURP1, CRP, VGF, WISP2, WISP1, WISP3, KLK12, KLK11, NRG2, APLN, CILP, GRP, OSM, AMH, CHGA, CFL2, ULBP2, CA6, WNT11, CA2, CTSG, CPO, CPM, SPOCK3, VPREB3, KRT9, CALCA, REN, PRSS2, COMP, KRT1, PTN, FGFBP2, MYOC, DPT, SCG2, KLK6, KLK7, SECTM1, KLK8, TNXB, KLK5, TNFSF9, A2ML1, IL36RN, GCG, KRT78, MEP1A, MEP1B, GPT, IGFBP1, CELA2A, GDF15, KLHL34, MUC16
GO:0005887~integral component of plasma membrane	247	1.86E-16	CADM3, SLC52A1, AQP9, CADM2, AQP8, AQP5, LPAR1, AQP6, AQP2, AGTR1, ATP2B3, AGTR2, ATP2B4, KCNK9, GRIN2B, ADAM2, NPSR1, HTR1D, HCAR3, CLCA1, CLCA4,

ABCB11, PTPRH, GRIN2A, HTR4, LIFR, TMEM130, CLEC4M, SSTR5, CCR8, SSTR2, VSIG2, SSTR3, CD36, RHCG, TMPRSS11D, TACSTD2, HTR7, TMPRSS11E, CCR2, TMPRSS11A, CALHM3, CLEC5A, PRLHR, CALY, FCER2, ADRB3, SLC11A1, MUSK, UPK1A, SLC28A1, SLC28A3, FCER1A, TRPC5, TRPC7, CELSR1, DCSTAMP, KCNK3, ATP13A4, EPHA5, CEL, SEMA6A, EPHA7, EPHA6, SEMA6D, EPHA8, AVPR1B, CHRNG, KCNJ16, KCNJ15, SLC15A1, TRPV3, SLC16A12, ANPEP, APCDD1, GPR87, GPR88, SLC26A2, KCNJ13, ASGR1, KISS1R, TNFRSF11B, SLC23A1, SLC22A3, MAS1L, TRPV6, CEACAM1, GHR, CACNG4, ACKR2, NECTIN4, GPER1, GRM4, SLC26A3, EREG, GRM8, SLC26A9, LRMP, GLRA4, KCNH8, SLC39A10, NPY2R, TFR2, BEST4, BEST2, BEST3, MAS1, FUT1, NPFFR1, SLC8A2, TRHDE, LRRN4, PTPRZ1, NLGN1, NPY1R, STAB2, P2RY12, EPGN, P2RY14, ADGRL3, HTR2C, CASR, SLC13A5, TSPAN1, SLC22A12, SLC6A20, GRIK3, GABRB1, SLC6A3, TSPAN7, TLR3, SLC7A9, SLC22A11, ENPEP, CNGB1, SLC7A5, GALR1, SLC2A4, SLC2A2, MS4A1, MS4A2, SLC4A4, RGR,

			SLCO4A1, MRGPRE, PROKR1, MRGPRF, CNGA3, GPR50, GPR52, PTGDR2, FXYD1, SLC38A5, FGFR2, SLC38A3, FXYD4, DRD2, ADCYAP1R1, DRD5, KCNA1, PTK7, KCNA5, SLC22A31, FXYD5, SLCO2A1, P2RY4, FOLR1, CNR1, P2RY1, CNR2, BTN1A1, GRPR, SCN9A, IFITM5, UPK2, GABRD, HCN1, TLR10, SLCO4C1, KLB, GABRA3, TNFRSF13B, SLC6A14, REEP2, SLC6A19, ABCB5, SLC4A10, LYVE1, SLC4A11, P2RX1, P2RX2, SLC13A1, ADRA1A, SLC13A2, CD79B, SLC13A3, CD79A, SCARA5, SLC5A11, SLC5A12, LRRC8E, CORIN, TACR2, LGR6, LGR5, CXCR5, GABRG2, NRXN1, NCR2, SLC7A14, UGT1A1, SLC7A11, TRPM1, SIGLEC6, CHRM2, CLDN1, CNTN2, GPR15, JAM2, GPR12, PLPP4, RHBG, OXTR, CLCNKB, GPR3, TPSG1, SLCO1A2, SCNN1G, SCNN1B, DCLK1, HPN, MPZ, OLR1, MAL, SLC10A2, RNF43, SLCO1B3, FZD10, SLC17A4, SLC17A1, SLCO1B1, SLC6A6, MEP1A, MEP1B, SLC18A3, SLC5A7, SLC14A2
GO:0005578~proteinaceous extracellular matrix	77	2.66E-16	CTHRC1, GLDN, WNT3A, MMP8, ENAM, MMP7, MMP28, MMP3, MMP1, SHH, CHAD, WNT2, OGN, MMP20, WISP2, TNFRSF11B, DSPP, WISP1,

			CILP2, WNT3, WISP3, CCBE1, TGFBI, COL11A1, COL10A1, MATN3, ADAMTS20, SPARCL1, CILP, MMP13, PRELP, SLIT3, MMP11, MMP10, ADAMTS6, AMBN, BGN, ANOS1, RELN, WNT11, EPYC, ADAMTS2, CHL1, ADAMTSL1, MAMDC2, SPOCK3, ADAMTSL3, VIT, TIMP1, COL9A3, HPSE2, COL6A6, CCL21, COL6A5, AMELX, RPTN, COMP, ACAN, ADAMTS12, MYOC, DPT, WNT8B, SBSPON, HAPLN1, TNXB, PTPRZ1, DMP1, CHI3L1, EMILIN3, COL4A6, LAMA1, WNT7B, OMD, COL19A1, SFRP1, ZG16, WNT7A
GO:0005198~structural molecule activity	69	7.13E-15	CLDN8, PRPH, CLDN9, CLDN6, LCE3D, KRT80, DES, KRT40, SNTB1, KRT222, KRT83, KRT13, EPB41L3, KRTAP3-1, CRCT1, PGM5, BVES, KRT17, KRT16, KRTAP3-2, KRT14, KRTAP3-3, OTOG, SPRR3, CLDN1, CLDN2, MAP7D2, SNTG2, CLDN16, KRT6C, KRT6A, CLDN18, SPRR2G, FLG2, SPRR2F, CLDN10, SPRR2E, CLDN11, CLDN14, KRT23, KRT24, KRT9, FGA, KRT5, SPRR2D, FGB, SPRR2A, KRT7, KRT1, KRT3, KRT4, IVL, NEFL, PRR9, NEFM, PLP1, MPZ, KRT34, KRT35, CLDN23, KRT32, KRT38, KRT75, KRT37, KRT74, LCE1E, SPRR1A, SPRR1B, KRT78
GO:0016324~apical	73	2.32E-12	ATP1B2, SLC22A12,

plasma membrane			SLC6A20, SLC9A3, AQP5, SLC7A9, SLC22A11, AQP6, ENPEP, PRKG2, SLC7A5, AQP2, KISS1, SLC23A1, ANK2, SLC2A2, TDGF1, NPC1L1, ATP6V0D2, DPEP1, CEACAM1, KCNMA1, TRPM6, CLCA4, CDHR2, GIF, CDHR5, IL6R, SLC26A3, CD36, RHCG, MGAM, OTOG, SLC26A9, CLDN1, CD300LG, CA4, STC1, CPO, SHROOM4, KCNA1, OXTR, CNTFR, NAALADL1, ATP6V1B1, GPR143, P2RY4, FOLR1, UPK1A, P2RY1, SCNN1G, SCNN1B, UPK2, NOX4, HPN, PLET1, CUBN, SI, MAL, GJB6, SLC10A2, ABCG8, ABCG5, SLC17A4, DSG1, P2RX2, SPTBN2, S100G, ATP6V0A4, LRP2, PDZK1, SLC14A2, SLC5A12
GO:0008544~epidermis development	33	2.27E-11	S100A7, LCE3D, SPRR2G, SPRR2F, SPRR2E, KRT9, DCT, COL7A1, KRT5, SPRR2D, SPRR2A, ZNF750, POU3F2, CALML5, KRT83, KLK7, NTF4, KLK5, KRT31, GRHL3, GJB5, EDAR, GRHL1, KRT34, KRT32, CST6, KRT17, LCE1E, SPRR1A, KRT16, SPRR1B, KRT14, SPRR3
GO:0000786~nucleosome	35	3.09E-11	HIST1H2AB, HIST1H4L, HIST1H2AD, HIST1H2BO, HIST2H2AB, HIST1H2BM, HIST1H4A, HIST1H2BL, HIST1H4B, HIST2H2AC, HIST1H2BI, HIST1H2BJ, HIST1H4E, HIST1H4F, HIST1H4C, HIST1H4D, HIST1H3J, HIST1H2BB,

			HIST1H1E, HIST1H1D, HIST1H2BE, HIST1H2BF, HIST1H1B, HIST1H1A, HIST2H3D, HIST1H3A, HIST1H3B, HIST1H2AH, HIST1H3C, HIST1H3D, HIST1H2AJ, HIST1H3F, HIST1H2AM, HIST1H2AL, HIST1H3I
GO:0007267~cell-cell signaling	60	1.64E-09	EDN3, INSL4, FCRL2, ENPEP, CXCL11, SHH, IL11, GJA3, PGR, WNT2, WISP2, WISP1, WISP3, NMUR2, GATA4, FGF3, CHST4, FGF21, INHA, FGF20, AMH, INHBA, SSTR2, SSTR3, EREG, SIGLEC6, GPR50, CARTPT, FGFR2, IL36G, PPY, CXCL5, CCL8, NMB, CXCL6, CCL26, CALCA, IL17C, PCSK1, ADRB3, IL17A, CCL23, INS, LHX1, CCL21, PCSK5, MLN, BMP3, TRHDE, NTF4, TBX5, TNFSF9, CCL13, LVRN, FJX1, ADRA1A, PYY, GDF15, SST, NR5A1
GO:0005179~hormone activity	31	5.89E-09	TG, VIP, EDN3, PPY, INSL4, INSL5, EDN2, CGB7, GAST, NMB, VGF, C1QTNF9, CALCA, INS, ERFE, APELA, GALP, GUCA2A, APLN, MLN, STC2, IGF2, INHA, INHBB, GCG, INHBA, AMH, UCN2, STC1, PYY, SST
GO:0005886~plasma membrane	529	3.54E-08	SLC9A9, SCN3A, SLC9A3, SLC9A2, FRRS1L, GNG8, AGTR1, ADTRP, AGTR2, MALL, DIRAS2, KCNK9, GRIN2B, GRIN2D, ADAM2, FAP, SERPINE1, GNG3, HTR1D, GNG4, VNN3, GNG7, CLCA1, CLCA4, GRIN2A, LIFR, CDHR5, F7,

SSTR5, SSTR2, SSTR3,
CD163L1, F5, F2, MGAM,
MADCAM1, SLC30A10,
LY6G6D, LY6G6F,
ARHGEF25, LY6G6C,
MAGEA1, SORCS3, ADRB3,
DUSP15, UPK1A, SLC28A1,
SLC28A3, PCDH10, IL1RN,
ATP11A, KCNK3, GNGT1,
PENK, PKP1, CD209,
AVPR1B, TREML4, CHRND,
RGS9, CHRNG, TRPV3,
PRSS41, UNC93A, NBEA,
LY9, GPR88, ST6GALNAC6,
SLC23A1, KISS1R, MAPT,
NMUR2, CEACAM7,
SLC22A3, MAS1L, STRA6,
TMEM100, TRPV6, KCNG3,
DPP6, DPEP1, CEACAM1,
GHR, PIK3C2G, CACNG8,
CACNG4, NECTIN4, IL6R,
CACNG1, FLNC, FLNA,
EPB41L3, FMN2, CARD14,
DOK7, CYBRD1, SERPINB2,
KCNH8, SCN11A, ARL4D,
KCNH4, NKD1, ALPI, FHL1,
NPY2R, GNG13, DISP2,
C2ORF88, ALPP, ITM2C,
CLEC10A, ITM2A, HTR3E,
IGSF11, SORBS1, HPSE2,
MAS1, PCSK9, KCNE2,
HTR3A, SNAP25, ACSL6,
HTR3C, ABCA12, SLC8A2,
IGF2, STAB2, NPY1R,
TMPRSS5, P2RY12, WNT7B,
CDH16, PTP4A3, PDE2A,
P2RY14, CDH19, GFRA1,
LRP8, LRP2, WNT7A,
HTR2C, CDH10, GFRA2,
GFRA3, SLCO1B7, TSPAN1,
SLC13A5, RAB9B,
SLC6A20, GRIK3, ZAN,
OR1J1, GRIK5, SLC7A9,
OR1J4, ENPEP, CYP2W1,

SLC7A5, RELL2, AZGP1, APOA1, ANK2, SLC2A4, GALR1, UNC5A, SLC2A2, TGFBI, C14ORF180, TDGF1, UNC5D, UNC5C, SLC51B, SLC51A, SIX2, RPH3A, NCAM1, TAS2R50, ANKRD13B, C2CD4B, RELN, C2CD4A, PTGDR2, ABCA8, DRD2, TNFRSF12A, DRD5, KCNA1, KCNA3, CNTFR, KCNA5, SLCO2A1, PDE6A, GPR22, AMER2, FGA, P2RY4, FGB, P2RY1, GRPR, OR51B5, PRIMA1, SH3TC2, GABRP, HCN1, GABRD, GABRE, TLR10, CUBN, SYT10, OR5M11, KCNB1, TNFRSF13B, SLC6A14, SLC6A19, ABCB5, LYVE1, DSG4, P2RX1, DSG3, DSG1, P2RX2, SLC13A1, LVRN, SLC13A2, ADAM20, ADRA1A, SLC13A3, SLC5A11, CLDN8, LRRC8E, CLDN9, CORIN, OR1N2, SDCBP2, OR2T10, RASL10B, NPC1L1, RAET1L, ANO5, TREH, SCN7A, NRG2, EVA1A, TRPM6, SGK1, EDAR, SIGLEC15, SLC7A11, TRPM1, TAS2R13, ULBP3, CA9, TAS2R19, CHRM2, ULBP1, CNTN2, GPR15, CLDN2, CNTN1, CA4, CNTN4, TREM1, CNTN3, CA2, JAM2, GAP43, PLPP4, PTCHD1, CLCNKB, OXTR, CDH3, CDH7, RGMA, ANXA8, SLCO1A2, TAS2R46, REN, DNER, TAS2R43, SCNN1G,

SCNN1B, SECTM1, PLP1,
VSTM2A, GNAO1, OLR1,
TNFSF9, XPNPEP2, GDPD2,
AJUBA, GCG, SLCO1B3,
PNOC, TAS2R38, SLCO1B1,
DSC3, SCN4B, MEP1B,
PLEKHN1, TAS2R30,
SLC14A2, SLC36A1,
CADM3, SYT4, AQP9,
GLDN, AQP8, CADM2,
ATP1B2, LYPD8, ADCY5,
FGFRL1, SYT8, AQP5,
SYT9, LPAR1, SHH, AQP2,
APOB, ATP2B3, ATP2B4,
CLEC4G, IZUMO1, NPSR1,
CHRNA6, HCAR3, MUC15,
HCAR2, HCAR1, ADGRB3,
PRAME, ABCB11, HTR4,
CCR8, CD36, RHCG, HTR7,
CCR2, OR2D2, CLEC5A,
ENPP6, ENPP7, PRLHR,
GSDMA, GSDMC, FCER2,
GPR143, KCNMB1,
CAMKV, SLC11A1, DMD,
GAD1, FCER1A, CNKSR2,
OR13H1, TRPC5, TRPC7,
CELSR3, CELSR1,
ATP13A4, ABCG2, EPHA5,
ABCG8, EPHA7, EPHA6,
ABCG5, SFRP1, SEMA6D,
EPHA8, ERVW-1, PDZK1,
PLAU, OR10A5, KCNJ16,
JPH4, KCNJ15, JPH2,
SLC15A1, OR10A2, TAS2R3,
FCRL2, FCRL1, SLC26A2,
FCRL4, TNFRSF11B,
FAM129A, KCNMA1,
PCDH11Y, ACKR1, CD160,
ACKR2, GPER1, KCNA10,
GRM4, SLC26A3, SGCG,
GRM8, SLC26A9, SLC27A6,
PLA2G3, ADAM12, CHL1,
PLA2G5, CLDN16, CLDN18,
LIMS2, SLC39A10, GPM6B,

			CLDN10, XKRX, ZBTB16, CLDN11, CLDN14, LY6D, GPM6A, CD177, CT83, PLIN4, CLEC2A, CD22, NPFFR1, CAP2, PIRT, SI, NLGN1, CBARP, GRIA4, ATP1A2, KCNV2, CLDN23, DKK1, KREMEN2, SLC16A9, ATP6V0A4, CASR, SLC22A12, SLC6A3, WNT3A, GABRB1, SLC22A11, CNGB1, WNT2, WNT3, MS4A2, GLP2R, SLC4A4, NEGR1, SLCO4A1, CA12, CHP2, TNFRSF17, MRGPRF, PROKR1, PLCE1, LYNX1, CD300LG, ANOS1, CD300LD, FGFR2, FXYD1, SLC38A5, SLC38A3, SNAP91, FXYD4, ADCYAP1R1, DSCAML1, FXYD6, FOLR1, CNR1, CNR2, ENTPD8, MSLN, ENTPD3, SLC30A8, AXIN2, TESC, SLCO4C1, KLB, GABRA3, CPNE7, SLC4A10, DIO2, CD79B, CD79A, TACR2, LGR6, LGR5, CXCR5, CNTNAP3, TAAR1, GABRG2, PRKCG, NRXN1, NCR2, PRKCB, BVES, PSD, CEMIP, CTSG, AOC3, CPM, PPP1R12B, RHBG, TRIB3, GSG1L, KRT5, KRT1, PALM2, PALM3, PLCD1, HPN, GPR119, CR2, MPZ, MUC21, SLC10A2, GPRC6A, RGS16, RGS13, SLC17A7, RNF43, RGS20, RGS21, SLC17A1, KCNN3, SLC6A6, SLC18A3, SLC5A7, MUC16
GO:0007155~cell adhesion	86	6.04E-08	SLURP1, ATP1B2, MYBPC3, MMRN1, CXCL12, HMCN2, AZGP1, WISP2, CGREF1,

			DSPP, WISP1, SRPX, WISP3, ADAM2, FAP, TGFBI, CNTNAP3, COL12A1, ROBO2, IZUMO1, CEACAM1, EGFL6, SIGLEC11, CDHR5, CHST4, NECTIN4, CTNNA3, NCAM1, CCR8, AMBN, PGM5, CD36, HEPACAM, SIGLEC6, SIGLEC8, CNTN2, ANOS1, CNTN1, RELN, MADCAM1, CNTN4, CNTN3, COL1A1, MFAP4, ADAM12, CHL1, AOC3, IBSP, CTNND2, PTK7, DSCAML1, CLDN10, CDH3, ITGBL1, IGSF11, LY6D, COL7A1, SORBS1, COL6A6, AMELX, COL6A5, COMP, MSLN, ACAN, CD22, THBS2, DPT, SPP1, HAPLN1, TNXB, OLR1, LRRN2, PCDH10, NLGN1, STAB2, COL4A6, LAMA1, LYVE1, OMD, COL19A1, CDH16, PKP1, EPHA8, DSC3, HABP2, MUC16
GO:0044267~cellular protein metabolic process	34	6.46E-08	ODAM, HIST1H4L, MMP1, APOA4, CALCA, APOA1, HIST1H4A, FGA, INS, HIST1H4B, TGFBI, SFTA3, HIST1H4E, SFTPC, HIST1H4F, HIST1H4C, HIST1H4D, SFTPB, NAT8, HIST1H3J, SEMG1, IGF2, MMP13, HIST2H3D, F2, HIST1H3A, HIST1H3B, HIST1H3C, HIST1H3D, PAPPA2, IGFBP1, HIST1H3F, CTSG, HIST1H3I
GO:0008083~growth factor activity	41	7.10E-08	FGF19, CXCL1, CSF2, FGF8, VGF, CXCL12, GDNF, MACC1, IL11, TIMP1, OGN, REG1A, WISP3, AMELX,

			TDGF1, PTN, ANGPTL3, NRG2, FGF3, BMP3, NTF4, NODAL, FGF23, IGF2, FGF21, INHA, FGF20, OSM, INHBB, AMH, INHBA, VEGFD, AMBN, DKK1, EREG, PPBP, EPGN, F2, GDF15, BMP7, BMP5
GO:0006508~proteolysis	91	9.77E-08	MASP1, CNDP1, PGC, PRSS41, MMP8, MMP7, MMP27, ANPEP, MMP28, MMP3, MMP1, CASP5, MMP20, ADAM2, FAP, DPEP3, KLK12, KLK11, KLK10, CPA1, PRSS33, DPP6, CFD, LONRF2, TMPRSS13, DPEP1, TMPRSS15, CELA3B, CLCA1, CLCA4, ADAMTS20, ELANE, PRSS50, COLEC10, CPXM2, MMP13, MMP11, MMP10, ADAMTS6, F5, TMPRSS11D, TMPRSS11E, F2, CAPN14, RELN, TMPRSS11A, ADAM12, PRSS48, CTSG, CPO, ADAMTSL1, ADAMTSL3, PRSS1, NAALADL1, TPSG1, PCSK2, PCSK1, REN, PRSS2, ACAN, ADAM33, FGL2, HGFAC, PRSS55, PRSS56, KLK7, CPA4, HPN, KLK8, TRHDE, OLR1, KLK5, FBXO2, KY, TMPRSS5, TMPRSS3, XPNPEP2, LCN1, SFRP1, MEP1A, LVRN, MEP1B, ADAM20, LRP8, PAPPA2, CELA2A, PRSS22, ADAMDEC1, PLAU, HABP2, TLL1
GO:0004252~serine-type endopeptidase activity	55	1.15E-07	CORIN, MASP1, PRSS41, MMP8, MMP7, MMP3,

			MMP1, MMP20, FAP, KLK12, KLK11, KLK10, PRSS33, CFD, TMPRSS13, TMPRSS15, CELA3B, PRSS50, ELANE, COLEC10, GZMB, F7, MMP13, MMP11, MMP10, F5, TMPRSS11D, TMPRSS11E, F2, TMPRSS11A, PRSS48, CTSG, PRSS1, TPSG1, PCSK2, PCSK1, PRSS2, PCSK9, HGFAC, PCSK5, PRSS55, KLK6, KLK7, PRSS56, KLK8, HPN, KLK5, TMPRSS5, TMPRSS3, CMA1, CELA2A, PRSS22, PLAU, HABP2, TLL1
GO:0007218~neuropeptide signaling pathway	30	2.15E-07	HCRT, PPY, PRLHR, NXPH4, NPY2R, SORCS1, NMB, GPR143, SORCS3, CALCA, KISS1R, GALR1, NMUR2, GALP, NPFFR1, NPSR1, NMU, UCN, PROKR1, NPY1R, GRP, SSTR2, NPY, PENK, PNOC, NPW, CARTPT, GLRA4, PYY, PTGDR2
GO:0030018~Z disc	33	3.17E-07	JPH2, PPP1R12B, PDLIM3, KCNA5, MYOT, MYL9, DES, ATP2B4, ANK2, DMD, NOS1, CRYAB, LDB3, KY, SYNPO2, MYH6, HOMER1, FLNC, NEXN, FLNA, NEBL, IGFN1, MYO18B, TRIM54, PGM5, NEB, CFL2, MYPN, RYR3, ADRA1A, FBXL22, CASQ2, AKAP4
GO:0006814~sodium ion transport	26	3.22E-07	SLC38A3, SLC13A5, SCN3A, ATP1B2, SLC38A8, SLC23A1, SCN9A, SCN7A, SCNN1G, SCNN1B, SLC4A4, SGK1, ATP1A2, SLC10A2, SLC17A8, SLC4A10, SLC4A11, SLC17A4,

			SLC13A1, SCN4B, SLC13A2, SCN11A, SLC13A3, SLC5A7, SLC5A11, SLC5A12
GO:0031225~anchored component of membrane	32	3.65E-07	LY6G6D, ENPP6, CPM, ALPI, LYPD8, ITLN1, LY6G6C, CNTFR, ALPP, RGMA, LY6D, CD177, TDGF1, MSLN, CEACAM7, DPEP3, RAET1L, NEGR1, DPEP1, VNN3, PLET1, XPNPEP2, NCAM1, LYNX1, CNTN2, CNTN1, CA4, GFRA1, CNTN4, CNTN3, GFRA2, GFRA3

KEGG pathway enrichment results for differentially expressed genes.

ID	Description	geneID
hsa04080	Neuroactive ligand-receptor interaction	GLP2R/PYY/GRIK3/GRIN2D/TACR2/SST/INSL5/EDN3/P2RY1/NPY2R/ADCYAP1R1/P2RY14/P2RX2/P2RX1/SSTR2/LPAR1/PPY/VIP/CNR1/EDN2/GRIK5/P2RY4/AGTR1/AVPR1B/NPY/APLN/CTSG/HTR7/THRB/GABRD/UCN2/GALR1/NMB/OXTR/GRIA4/GRIN2B/HTR4/ADRA1A/NPFFR1/GHR/CHRM2/UCN/SSTR3/PENK/CNR2/GABRE/HTR1D/GRIN2A/GABRG2/ADRB3/NPY1R/SSTR5/NMU/KISS1/GCG/F2/NPW/PRSS1/CALCA/HCRT/KISS1R/GABRP/DRD5/DRD2/GRPR/GRM8/CHRNA6/PRSS2/GRP/CHRND/CHRNG/AGTR2/NMUR2/HTR2C/GABRB1/MAS1/CALCB/GRM4/PRLHR/TAAR1/GABRA3/GPR50/MLN
hsa05034	Alcoholism	CREB3L3/GNAO1/GRIN2D/GNG7/ADCY5/GNG3/NPY/GRIN2B/GNG8/MAOB/GRIN2A/TH/HIST1H3J/HIST1H4C/HIST1H2AH/HIST1H4E/HIST1H4B/HIST1H4D/GNG13/HIST3H2BB/HIST1H3B/HIST1H2BO/HIST2H2BF/HIST1H2AB/HIST1H2AL/HIST1H3I/HIST1H2BI/HIST1H2AJ/DRD2/GNGT1/HIST2H2AB/GNG4/HIST1H2BL/HIST1H2BE/HIST1H4L/HIST1H2BF/CALML3/HIST2H2AC/HIST1H2AM/HIST1H4A/HIST1H2AD/HIST2H3D/HIST1H3F/HIST1H2BB/HIST1H2BM/HIST1H3D/HIST1H2BJ/SLC6A3/HIST1H3A/HIST1H3C/CALML5/HIST1H4F
hsa0532	Systemic lupus eryt	ELANE/C7/CTSG/GRIN2B/GRIN2A/HIST1H3J/HIST1H4C/HIST1H2AH/HIST1H4E/HIST1H4B/HIST1H4D/HIST3H2BB/HIST1H3B/HIST1H2BO/HIST2H2BF/HIST1H2AB/HIST1H2AL/HIST1H3I/HIST1H2BI/HIST1H2AJ/HIST2H2AB/HIST1H2BL/HIST1H2BE/HIST1H4L/HIST1H2BF/HIST2H2AC/HIST1H2AM/HIST1H4A/HIST1H2AD/HIST2H3D/HIST1H3F/HIST1H2BB/HIST1H2BM/HIST1H3D/HIST1H2BJ/HIST1H3A/HI

2	hematosus	ST1H3C/HIST1H4F
hsa04976	Bile secretion	SLC51A/ABCG2/CA2/AQP8/SLC51B/ABCB11/SLC4A4/ATP1A2/CYP3A4/SLC10A2/SLC9A3/ADCY5/NR1H4/ATP1B2/SLCO1B3/ABCG5/SULT2A1/BAAT/ABCG8/SLCO1B1/SLCO1B7/SLCO1A2/AQP9/UGT2B4
hsa04970	Salivary secretion	PRKG2/ATP1A2/ADCY5/ATP2B4/KCNMA1/ATP1B2/BEST2/PRKCB/CST1/RYR3/CST2/NOS1/ADRA1A/CST4/LPO/ADRB3/PRKCG/AQP5/CST5/ATP2B3/CALML3/PRB1/CALML5/PRB2/PRH2/TRPV6/MUC7
hsa04975	Fat digestion and absorption	APOA1/APOB/APOA4/FABP2/MOGAT2/PLA2G2C/PLA2G5/FABP1/CD36/CEL/ABCG5/NPC1L1/ABCG8/PLA2G3/PLA2G2F/LIPF/PNLIP
hsa00982	Drug metabolism - cytochrome P450	GSTA2/UGT1A8/HPGDS/GSTM5/CYP3A4/UGT1A10/ADH1C/GSTA1/ADH1B/FMO5/UGT2B15/UGT2B17/CYP2D6/UGT2A3/MAOB/UGT1A1/UGT1A5/ALDH3B2/ADH1A/UGT1A9/UGT1A3/UGT1A4/UGT2B4
hsa04977	Protein digestion and	MEP1B/SLC36A1/ATP1A2/SLC6A19/SLC9A3/COL11A1/ATP1B2/SLC7A9/COL10A1/MEP1A/COL7A1/SLC8A2/SLC15A1/COL4A6/COL6A5/COL1A1/XPNPEP2/COL12A1/COL9A3/KCNJ13/CELA3B/PRSS1/PRSS2/COL6A6/CELA2A/CPA1

4	absorption	
hsa05204	Chemical carcinogenesis	SULT1A2/GSTA2/UGT1A8/HPGDS/GSTM5/CYP3A4/UGT1A10/ADH1C/GSTA1/ADH1B/SULT1A1/UGT2B15/UGT2B17/UGT2A3/CYP2C18/UGT1A1/UGT1A5/ALDH3B2/ADH1A/UGT1A9/SULT2A1/UGT1A3/UGT1A4/UGT2B4
hsa00830	Retinol metabolism	UGT1A8/CYP3A4/UGT1A10/RDH5/ADH1C/ADH1B/DHRS9/UGT2B15/UGT2B17/LRAT/UGT2A3/CYP2C18/UGT1A1/UGT1A5/RDH16/ADH1A/UGT1A9/UGT1A3/UGT1A4/CYP26A1/UGT2B4
hsa00980	Metabolism of xenobiotics by cytochrome P450	GSTA2/UGT1A8/HPGDS/GSTM5/CYP3A4/UGT1A10/ADH1C/GSTA1/ADH1B/UGT2B15/UGT2B17/CYP2D6/UGT2A3/UGT1A1/UGT1A5/ALDH3B2/ADH1A/UGT1A9/SULT2A1/UGT1A3/UGT1A4/UGT2B4
hsa00140	Steroid hormone biosynthesis	UGT1A8/CYP3A4/DHRS11/UGT1A10/HSD3B2/HSD17B2/HSD11B2/UGT2B15/UGT2B17/UGT2A3/SULT2B1/AKR1C4/UGT1A1/UGT1A5/UGT1A9/UGT1A3/UGT1A4/CYP19A1/UGT2B4
hsa0	Ascorbate	UGT1A8/UGT1A10/UGT2B15/UGT2B17/UGT2A3/UGT1A1/UGT1A5/UGT1A9/MIOX/UGT1A3/UGT1A4/UGT2B4

0053	and aldarate metabolism	
hsa04978	Mineral absorption	TRPM6/ATP1A2/MT1M/MT1F/SLC26A3/SLC6A19/MT1G/MT1H/SLC9A3/MT1X/ATP1B2/MT2A/CYBRD1/MT1E/SLC26A9/S100G/TRPV6
hsa00040	Pentose and glucuronate interconversions	UGP2/UGT1A8/UGT1A10/UGT2B15/AKR1B10/UGT2B17/UGT2A3/UGT1A1/UGT1A5/UGT1A9/UGT1A3/UGT1A4/UGT2B4
hsa04060	Cytokine-cytokine receptor interaction	IL6R/BMP3/LIFR/CCL23/CXCL12/TNFRSF13B/INHBA/TNFRSF17/CCL14/CNTFR/TNFRSF12A/GDF15/CCL28/BMP5/IL11/CCR2/CCL8/CXCL3/GHR/CCL13/CXCL1/TNFSF9/CXCR5/IL23A/AMH/CCL21/CXCL2/CCL19/CXCL8/BMP7/IL1A/CXCL5/CSF2/PPBP/EDAR/CXCL17/CCR8/IL1RN/IL17C/CXCL6/OSM/INHBB/TNFRSF11B/CXCL11/CCL26/IFNE/IL36RN/INHA/NODAL/IL36G/IL17A/IL17F/IL36B/IFNK
hsa04742	Taste transduction	SCNN1B/SCN9A/P2RY1/SCNN1G/P2RX2/P2RY4/HTR3E/SCN3A/HTR3A/HTR1D/TAS2R38/GNG13/HTR3C/TAS2R30/TAS2R19/TAS2R3/TAS2R13/GRM4/TAS2R46/TAS2R43/TAS2R50/GABRA3

hsa04972	Pancreatic secretion	CA2/SLC4A4/ATP1A2/SLC26A3/CLCA4/ADCY5/ATP2B4/KCNMA1/ATP1B2/PRKCB/PLA2G2C/PLA2G5/CEL/PRKCG/CELA3B/PLA2G3/PRSS1/CLCA1/ATP2B3/PRSS2/PLA2G2F/CELA2A/PNLIP/CPA1
